# Adult Intramedullary Ewing Sarcoma of the Proximal Hip

**DOI:** 10.1155/2014/916935

**Published:** 2014-06-12

**Authors:** Preetam Gongidi, Siva Jasti, William Rafferty, Veniamin Barshay, Richard Lackman

**Affiliations:** ^1^Department of Radiology, Cooper University Hospital, Cooper Medical School of Rowan University, One Cooper Plaza B23, Camden, NJ 08103, USA; ^2^Department of Pathology, Cooper University Hospital, Cooper Medical School of Rowan University, One Cooper Plaza B23, Camden, NJ 08103, USA; ^3^MD Anderson Cancer Center at Cooper Orthopedic Oncology Center, Cooper University Hospital, Cooper Medical School of Rowan University, One Cooper Plaza, B23, Camden, NJ 08103, USA

## Abstract

Ewing sarcoma of bone is classically a permeative lesion in the diaphysis of long bones in children. While they occur primarily in children and adolescents, they can be seen in young adults in their 20s, but these are typically seen in flat bones. The permeative nature of the lesion can elicit new bone formation creating a partially sclerotic appearance, cortical expansion presenting as a “Codman triangle,” or have an “onion-skin” type of aggressive periosteal reaction/periostitis. Ewing sarcoma is rarely seen without an associated soft-tissue mass and is even rarer to just have benign-appearing periostitis (e.g., thick, uniform, or wavy cortex). We present such a case of Ewing sarcoma in a young adult confined to just the medullary metadiaphysis without cortical erosion or soft-tissue mass. To the best of our knowledge, this is the first case to be reported in the radiology literature.

## 1. Case Report

**Figure 1:**
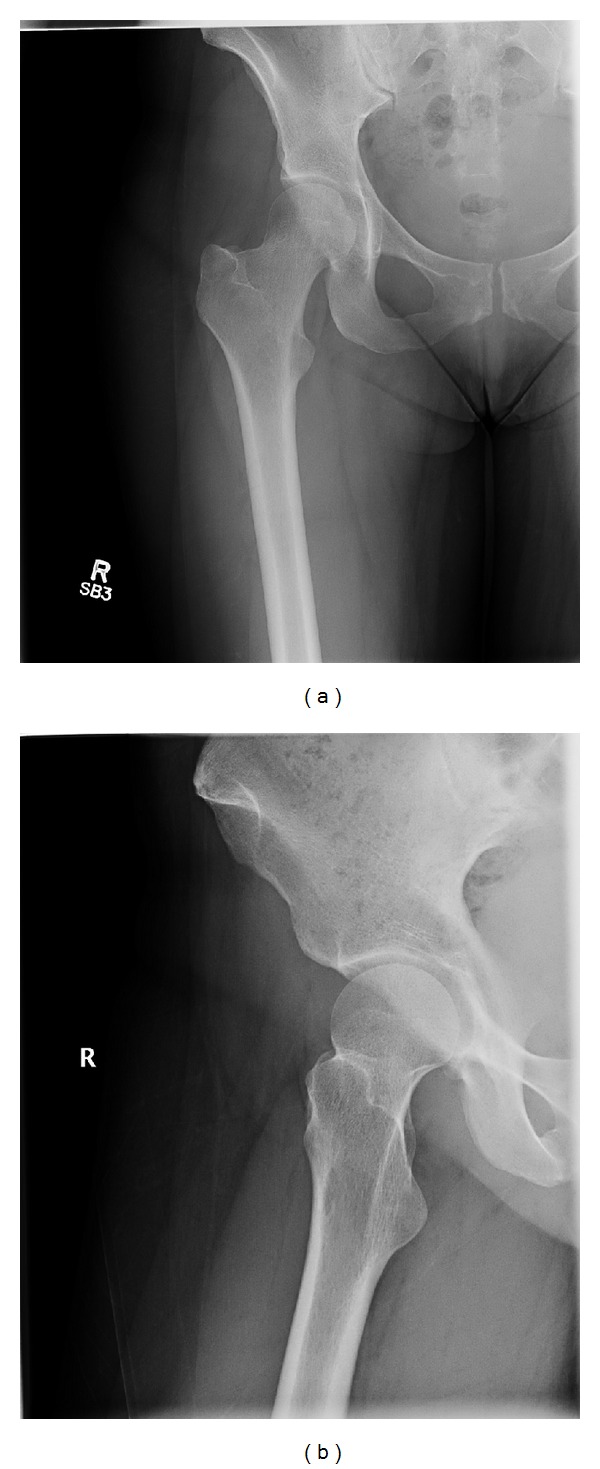
Frontal (a) and internal (b) rotation plain radiographs of the right hip reveal no discrete osseous lesion, cortical destruction, or soft-tissue mass.

We present a 29-year-old postpartum female with a four-month history of right groin pain that occasionally radiated into the hip and anterior right thigh. The pain was intermittent but worsening. She decided on physical therapy but with no improvement. Her medication was limited to ibuprofen as she was still breastfeeding and the pain worsened with standing and lifting her baby. She denied history of trauma, fever, chills, night sweats, weight loss, or night pain. Plain radiographs of the right hip and femur were normal (Figure 1).

**Figure 2:**
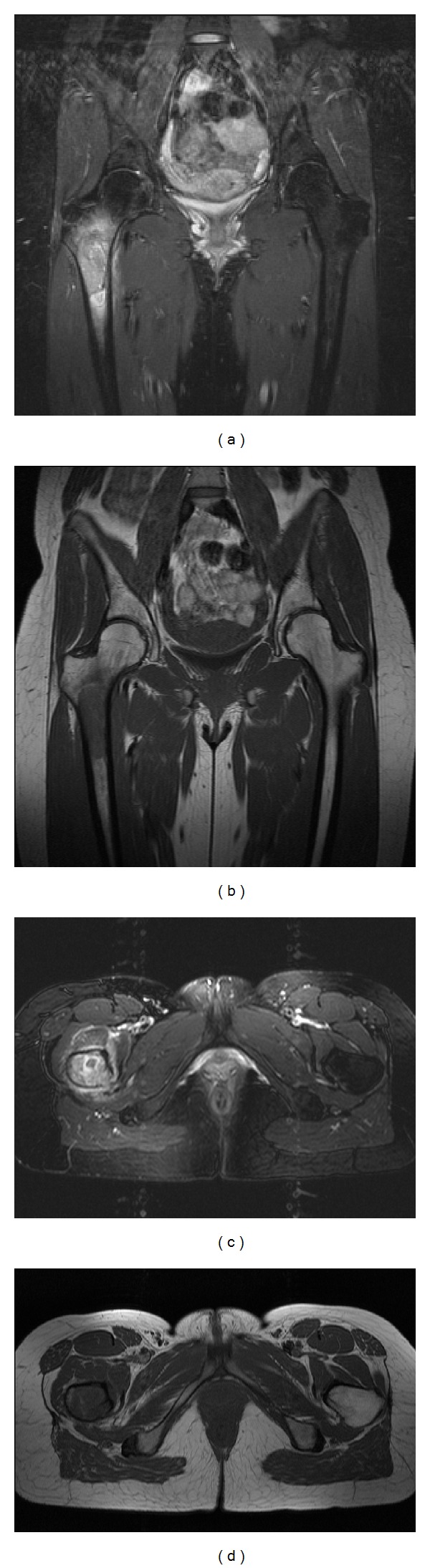
Unenhanced MRI of the right hip reveals a hyperintense lesion on STIR sequence ((a) coronal and (c) axial) and a correspondingly hypointense lesion on TI-weighted sequence ((b) coronal and (d) axial) with minimal cortical bone changes and no soft-tissue mass.

**Figure 3:**
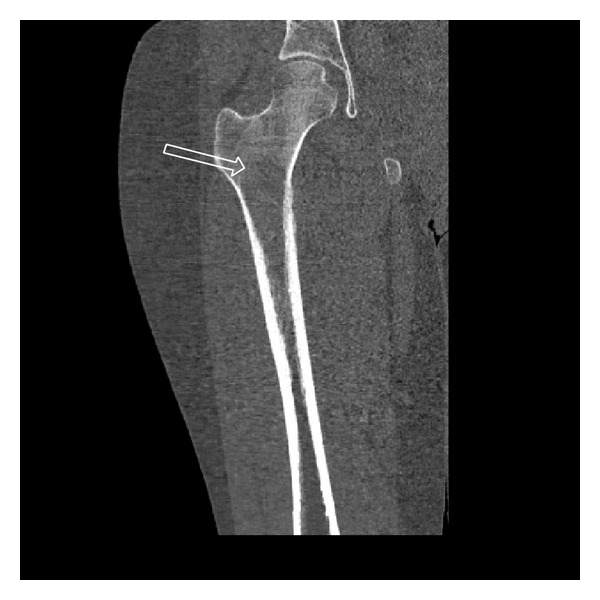
Coronal CT reformation of the right proximal femur reveals subtle low-attenuating lesion without cortical destruction or soft-tissue component. The arrow points to the superior margin of the ill-defined lesion.

She subsequently received an MRI of the right hip which revealed a large marrow-replacing lesion extending from the intertrochanteric region into the proximal diaphysis with surrounding edema in the bone and soft tissues (Figure 2). The patient was emergently admitted for an impending pathologic fracture. The CT of the right hip revealed a focal bone lesion involving the right intertrochanteric region and proximal right femoral diaphysis with cortical irregularity (Figure 3).

**Figure 4:**
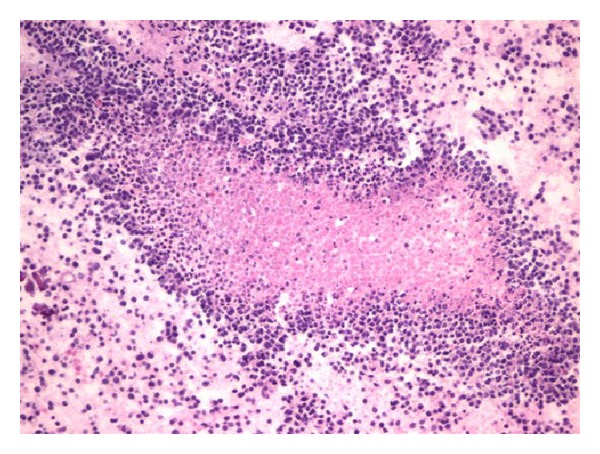
Cell block reveals viable small round cells surrounding a central focus of necrosis.

**Figure 5:**
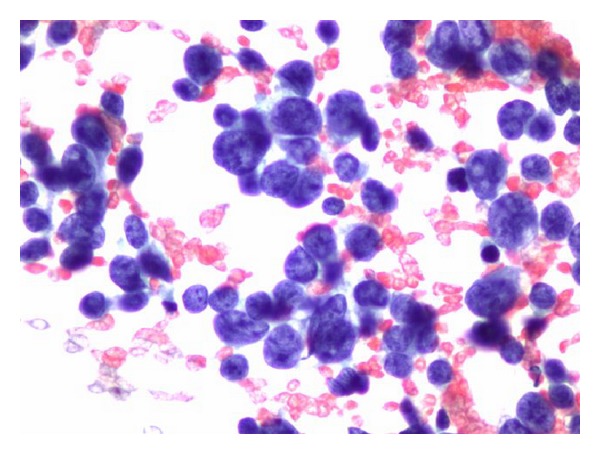
Small dark relatively uniform nuclei with indistinct cytoplasm.

**Figure 6:**
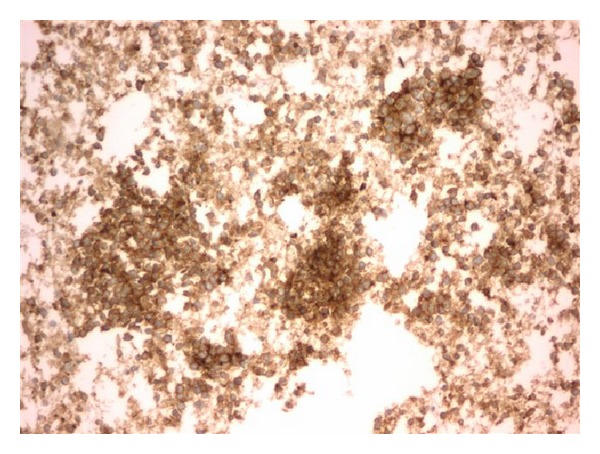
Cytoplasmic reactivity for CD99.

**Figure 7:**
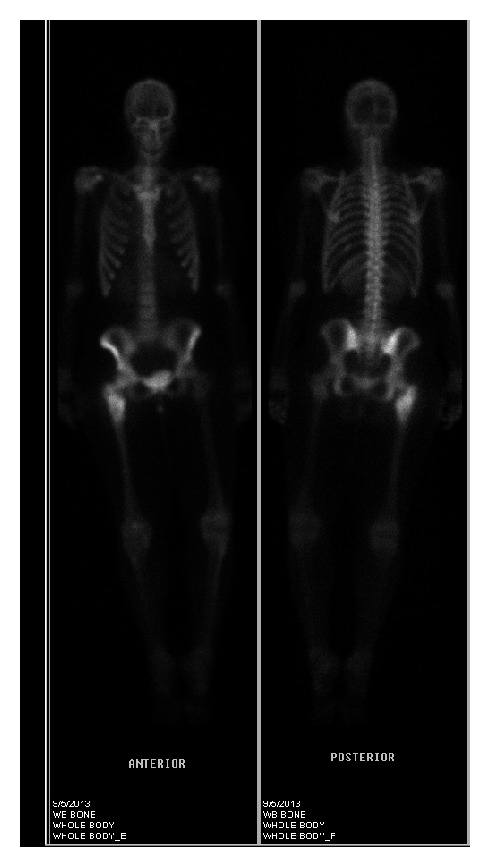
Whole body bone scan utilizing Tc-99 m MDP reveals a discrete area of increased radiotracer uptake within the metaphysis of the right proximal femur. No additional areas of abnormal radiotracer uptake are seen.

**Figure 8:**
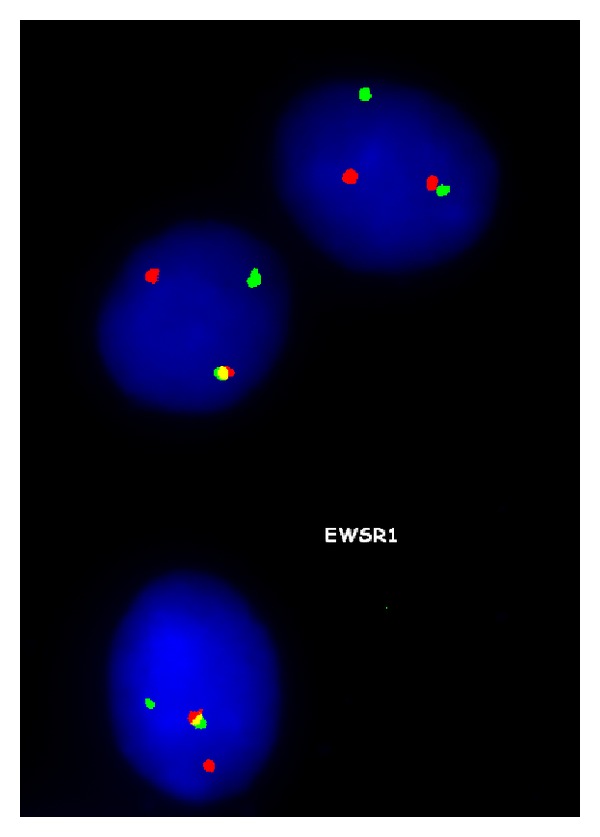
FISH study reveals the translocation involving EWSR1 gene at 22q12 with separate red and green signals compatible with Ewing sarcoma. As a side note, dual-color probes are located on either side of the EWSR1 gene and are normally seen as a single fusion signal.

With a working diagnosis of lymphoma, the fine-needle biopsy of the lesion instead revealed viable small round blue cells surrounding a central focus of necrosis (Figure 4) and small dark relatively uniform nuclei with indistinct cytoplasm (Figure 5). The immunoperoxidase staining was positive for CD99 (Figure 6) by microscopic examination. Leukemia and lymphoma cell markers were negative. The whole body bone scan revealed distinct high radiotracer uptake in the proximal right femur without evidence of metastasis (Figure 7). Further testing included fluorescence in situ hybridization (FISH) study which tested positive for the rearrangement involving EWSR1 gene at chromosome 22q12 (Figure 8) and was diagnostic for Ewing sarcoma.

**Figure 9:**
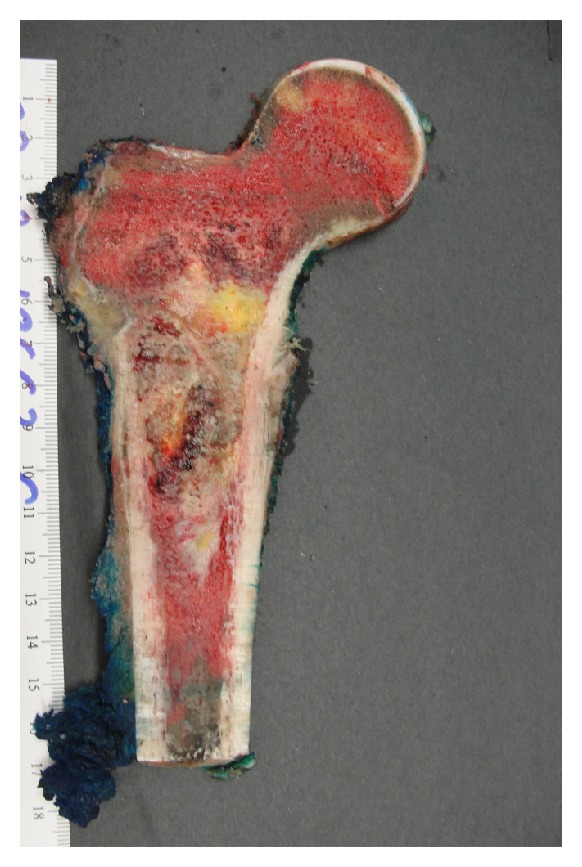
Gross specimen of the right proximal femur with wide resection of the 7.5 cm tumor reveals no viable tumor after chemotherapy.

The patient underwent chemotherapy followed by surgical wide resection and prosthetic reconstruction. The resected gross specimen showed no residual viable tumor by microscopic examination (Figure 9).

## 2. Discussion

Ewing sarcoma (ES) is one entity of what is now considered to be a group termed Ewing sarcoma family tumors (ESFT), which includes peripheral primitive neuroectodermal tumors (PNETs) and Askin tumors [1]. ES accounts for less than 10% of all primary bone tumors, is aggressive, and is considered a high-grade one. It is the second most common primary bone tumor in children and adolescents, typically between the ages from 5 to 20 years [1, 2].

The characteristic radiologic appearance of ES is a permeative osteolytic lesion involving the diaphysis or less commonly the metaphysis of a long bone but also occurs in the flat bones. The clinical course of the tumor when it is metaphyseal begins as an intramedullary lesion that causes marrow replacement and permeates through the cortical bone and extends into the adjacent soft tissues. Interestingly, the diaphyseal course also begins in the intramedullary canal but then permeates the cortex via haversian canals and vascular channels and extends into the surrounding soft tissue leaving behind the more characteristic periosteal, multilayered reactive bone formation commonly described as an “onion-skin” periosteal reaction [1]. Our case study is unusual because of not only the age of presentation but also its predominant metaphyseal location and intramedullary subsistence. This was a more characteristic presentation for lymphoma. It is our hope that readers will use this case to underscore the importance of keeping an appropriate differential diagnosis while at the same time realizing how a minimally invasive fine-needle aspiration biopsy can yield a definitive diagnosis. A study by Häussler et al. looked at cortical bone abnormality on MRI to help distinguish between primary lymphoma and osteosarcoma and Ewing sarcoma. They suggest that the homogeneity of the tumor may be a helpful criterion to distinguish lymphoma from sarcoma but quickly admit its contradiction to the current literature. They postulate that a bone lesion with an extraosseous component and minimal or no cortical changes is suggestive of lymphoma [3]. Our case study had these very similar characteristics.

Osseous lymphoma can be confused with Ewing sarcoma not only clinically but also pathologically. Ewing sarcomas microscopically appear as sheets of small blue round monomorphic cells with little intercellular stroma [4]. Several histologic variants are recognized, thus making definitive diagnosis incomplete. Immunohistochemistry offers a slightly more reliable diagnostic aid such as staining for vimentin and CD99 but recent data has shown that CD99 may also be positive in B-cell lymphoblastic lymphoma [5]. The genetics of the tumor offers a more definitive diagnosis with chromosomal translocation involving chromosome 22, particularly the t(11; 22) (q24; q12) translocation accounting for approximately 90% of the tumors [2, 4].

In summary, Ewing sarcoma is a very aggressive tumor with variable prognosis depending on stage, site, size, microscopic feature, and biologic features. Prognosis has improved thanks to modern chemotherapy advancements.
